# Identification of cancer stem cell characteristics in liver hepatocellular carcinoma by WGCNA analysis of transcriptome stemness index

**DOI:** 10.1002/cam4.3047

**Published:** 2020-04-20

**Authors:** Kun‐Hao Bai, Si‐Yuan He, Ling‐Ling Shu, Wei‐Da Wang, Shi‐Yong Lin, Qian‐Yi Zhang, Liang Li, Lei Cheng, Yu‐Jun Dai

**Affiliations:** ^1^ Department of Endoscopy Sun Yat‐Sen University Cancer Center Guangzhou China; ^2^ State Key Laboratory of Oncology in South China Guangzhou China; ^3^ Collaborative Innovation Center for Cancer Medicine Guangzhou China; ^4^ The University of Texas MD Anderson Cancer Center UTHealth Graduate School of Biomedical Sciences Houston TX USA; ^5^ Department of Hematological Oncology Sun Yat‐Sen University Cancer Center Guangzhou China; ^6^ Collaborative Innovation Center for Cancer Medicine Cancer Institute Fudan University Shanghai Cancer Center Shanghai China

**Keywords:** biomarker, cancer stem cells (CSCs), co‐expression network, liver hepatocellular carcinoma (LIHC), mRNAsi index, WGCNA analysis

## Abstract

Cancer stem cells (CSCs) are characterized by self‐renewal and ‐differential potential as compared to common cancer cells and play an important role in the development and therapeutic resistance of liver hepatocellular carcinoma (LIHC). However, the specific pathogenesis of LIHC stem cells is still unclear, and the genes involved in the stemness of LIHC stem cells are currently unknown. In this study, we investigated novel biomarkers associated with LIHC and explored the expression characteristics of stem cell‐related genes in LIHC. We found that mRNA expression‐based stemness index (mRNAsi) was significantly overexpressed in liver cancer tissues. Further, mRNAsi expression in LIHC increased with the tumor pathological grade, with grade 4 tumors harboring the greatest stem cell features. Upon establishing mRNAsi scores based on mRNA expression of every gene, we found an association with poor overall survival in LIHC. Moreover, modules of interest were determined based on weighted gene co‐expression network analysis (WGCNA) inclusion criteria, and three significant modules (red, green, and brown) and 21 key genes (*DCN*, *ECM1*, *HAND2*, *PTGIS*, *SFRP1*, *SRPX*, *COLEC10*, *GRP182*, *ADAMTS7*, *CD200*, *CDH11*, *COL8A1*, *FAP*, *LZTS1*, *MAP1B*, *NAV1*, *NOTCH3*, *OLFML2A*, *PRR16*, *TMEM119*, and *VCAN*) were identified. Functional analysis of these 21 genes demonstrated their enrichment in pathways involved in angiogenesis, negative regulation of DNA‐binding transcription factor activity, apoptosis, and autophagy. Causal relationship with proteins indicated that the Wnt, Notch, and Hypoxia pathways are closely related to LIHC tumorigenesis. To our knowledge, this is the first report of a novel CSC biomarker, mRNAsi, to predict the prognosis of LIHC. Further, we identified 21 key genes through mRNA expression network analysis, which could be potential therapeutic targets to inhibit the stemness of cancer cells in LIHC.

## Graphical abstract

We first reported a novel biomarker mRNAsi of stem cell index to predict the prognosis of LIHC through network analysis of the expression levels of LIHC. 21 key genes were found played important roles in LIHC stem cells. These genes could be the potential therapeutic targets for inhibiting the stemness of LIHC.

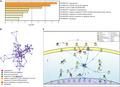

## 1 INTRODUCTION

Liver cancer is the fifth most common cancer worldwide, with a high mortality rate.^1^, ^2^, ^3^ However, the biological mechanism of hepatocarcinogenesis is still unclear. In recent years, the theory of tumor stem cells has been recognized. Tumor stem cells have the potential of self‐renewal and plasticity, and they play a decisive role in the initiation of tumor formation and growth.^4^, ^5^ Tumor stem cells exhibit strong characteristics of drug resistance and metastasis, thus existing treatments are ineffective, which may be the main reason for the recurrence and drug resistance of liver cancer.^6^, ^7^

Regarding a mechanism, Zusen et al identified noncoding RNAs that regulate the maintenance of liver cancer stem cells (CSCs) and found that C8orf4 could interact with N2ICD to inhibit the Notch signaling pathway and negatively regulate the self‐renewal of liver CSCs.^8^ Vitamin C can lead to DNA damage and energy depletion by increasing reactive oxygen species in cells, killing CSCs preferentially and improving patient prognosis.^9^ In addition, it can be combined with traditional platinum chemotherapy drugs to increase the sensitivity of tumor cells to chemotherapy drugs.^10^ However, the specific pathogenesis of LIHC stem cells is still unclear.

In recent years, with the rapid development of multigenomic sequencing, the genome, epigenome, transcriptome, and proteome characteristics of tumor cells are considered to be closely related to the dedifferentiation (loss of differentiated phenotypes) and stem cell acquisition of cancer cells.^11^ The molecular maps of embryonic stem cells and tumor cells were compared by machine learning algorithm. These algorithms analyzed a large number of data through advanced statistical techniques to explore patterns that can be used for decision‐making or prediction. Stem cell index is a new index to measure tumor development. The mRNA expression‐based stemness index (mRNAsi) and DNA methylation‐based stemness index (mDNAsi) scores of three types of poorly differentiated tumors (breast cancer, acute myeloid leukemia, and glioma) indicated that mRNAsi score is closely related to the biological process of CSCs.^11^ Some researchers found that metastatic tumors had higher stem cell indexes as well as a negative correlation between stem cell index and survival rate.^12^ The researchers found that in some tumor types, high stem cell index was associated with mutation. For example, in squamous cell carcinoma of the head and neck, high stem cell index was found to be related to *NSD1* gene mutation.^13^ In addition, it could recognize molecules associated with the dedifferentiation of certain tumors. The increase in FOXM1 protein levels is related to decreased cell differentiation and increased cell proliferation in breast and lung cancers.^14^, ^15^ Further, decreased expression of ANEXIN‐A1 protein in lung adenocarcinoma is related to an increased stem cell index.^16^

At present, the stem cell index is used as a prognostic indicator in few tumors to help predict the risk of tumor recurrence and guide treatment. To evaluate the role of mRNAsi in liver hepatocellular carcinoma (LIHC), we applied a special approach to identify stemness‐related genes through WGCNA. Taken together, these results might provide novel insights into the roles of some stemness‐related genes in LIHC.

## 2 MATERIALS AND METHODS

### 2.1 Data preparation

The RNA‐sequencing (RNA‐seq) results of 50 normal tissues and 374 cases of human LIHC samples and the related clinical data from 363 cases were obtained from the TCGA database (https://tcga‐data.nci.nih.gov/tcga/). These data were current as of 5 December 2019. We used a merge script by Perl to combine the RNA‐seq data of the 374 cancer samples into a signal matrix file. Next, we converted the related gene names from Ensembl IDs to corresponding gene symbols in the Ensembl database. In addition, the continuously updated clinical data in TCGA database were downloaded and filtered for useful information as well as the information of sex, age, life‐status, and stages. The survival time of patients is more accurate than that from other sources. Furthermore, the study design was exhibited in a flow diagram (Figure S1).

### 2.2 mRNAsi in modules

mRNAsi is an index that describes the similarity between cancer cells and stem cells and it might be considered a quantitative form of CSCs. The metastatic tumors had higher stem cell indexes as well as a negative correlation between stem cell index and survival rate. A comprehensive molecular feature was performed using LIHC samples from TCGA as described previously to obtain the detailed molecular subtyping of each sample and the correlation index with mRNAsi.^11^, ^12^ Kruskal‐Wallis analysis was conducted to verify significant differences among these modules. Samples were divided into two groups according to mRNAsi scores, and prognosis evaluation of mRNAsi scores was performed based on overall survival (OS) analysis by GraphPad Prism 8. Statistical significance was calculated by logrank tests and unpaired *t* test.

### 2.3 Differentially expressed genes

The “edgeR” R package was utilized to compare expression levels between normal tissues and tumor samples. Further, differentially expressed genes (DEGs) were selected using the following criteria: (a) fold change > 2; (b) false discovery rate (FDR) < 0.05; and (c) gene expression levels > 1.

### 2.4 WGCNA analysis

The WGCNA R package was used to perform the subsequent analyses.^17^ We selected the highest 25% of DEGs variance to ensure heterogeneity and accuracy of bioinformatics for co‐expression network analysis. First, the outliers in RNA‐seq data were filtered. Subsequently, the co‐expression analysis was constructed for paired genes using a Pearson correlation matrix. Next, the weighted adjacency matrix was constructed using the power function a_mn_ = |c_mn_|b, as previously described.^18^ We selected an appropriate b value to enhance the matrix similarity and construct a co‐expression network. We further converted the adjacency matrix into a topological overlap matrix (TOM) to detect gene connectivity in the network. Finally, an average linkage hierarchical clustering was performed based on TOM‐based dissimilarity, with a gene dendrogram over 30 to construct module dendrograms for further analysis.

### 2.5 Identification of significant modules and key genes

To determine the importance of each module, we calculated gene significance between the sample traits and gene expression as previously reported.^12^ In the principal components analysis of each module, the main components were composed of the module eigengenes (MEs) with an expression profile signature summarized by the expression patterns of all genes. The regression relationship between clinical data and gene expression was based on the *P*‐value. To enhance the productivity of the modules, a cutoff (<0.25) was selected to combine similar modules. mRNAsi and epigenetically regulated mRNAsi (EREG‐mRNAsi) were used as clinical phenotypes and further analyzed clinical phenotype with the gene modules. The statistical significance was calculated as *P* values (*P* < .05). Further, the correlation between genes in corresponding modules and gene expression profiles was defined as module membership (MM). Once the modules of interest were selected, gene significance (GS) and MM were determined for each important gene, and thresholds of cor. gene MM > 0.8 and cor. gene GS > 0.5 were established to screen key genes in each module.

### 2.6 Gene co‐expression analysis

The R corrplot package was utilized to analyze the co‐expression relationships between candidate genes by Pearson's correlation analysis.^19^ The strength of the relationships was calculated by gene expression levels.

### 2.7 Oncomine database analysis

An online cancer microarray database Oncomine was used to analyze gene expression levels of selected genes in the red, green, and brown modules of different cancer type and normal samples. Compared to the paired normal controls, differences in the transcriptome level of candidate genes in clinical cancer specimens were determined by Student's *t* test.

### 2.8 Causal relationship and protein interactions

Causal interaction data, which were annotated in SIGNOR, and protein interaction information in Mentha were used to investigate the protein interaction network associated with disease genes with the DisNor website.^20^ The first neighbor was selected to analyze protein relationships at the complexity level, and level 3 of the multi‐protein module was used to analyze the detailed protein interaction network among key genes.

### 2.9 Metascape analysis

Comprehensive functional analysis of candidate genes was performed by Metascape,^21^ which clustered all enriched functional terms into nonredundant groups. Next, the MCODE algorithm was applied to find the densely connected complexes for all components with alternations for performance improvements. Cytoscape was used to generate the visualized network result.

## 3 RESULTS

### 3.1 The mRNAsi and clinical characteristics in LIHC

**FIGURE 1:**
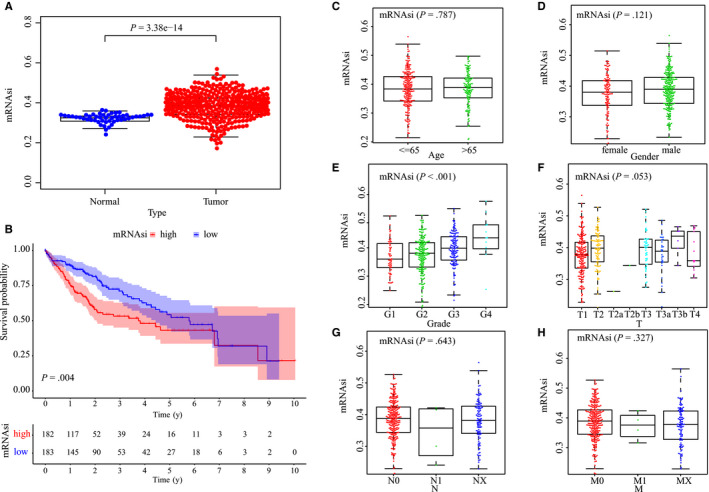
The mRNAsi and clinical characteristics in liver hepatocellular carcinoma (LIHC). A, Expression level of mRNAsi in normal (50 samples) and tumor (374 samples) tissues. B, Kaplan‐Meier survival analysis of relationship between mRNAsi and survival time. Comparison between mRNAsi expression level and clinical characteristics in LIHC, including age (C), gender (D), pathological grade (E), and TNM stage (F‐H)

mRNAsi can be used as an indicator to assess similarity and heterogeneity between malignant cells and stem cells, in order to estimate the number of CSCs. The mRNAsi algorithm is based on the molecular features of normal cells possessing differences in stemness potential. To explore mRNAsi in LIHC, we compared the expression of mRNAsi between normal and tumor tissues using the TCGA dataset. As shown, a significantly higher expression level of mRNAsi was identified in tumor tissues than normal samples (*P* < .001; Figure 1A). Next, the mRNAsi crucial potency for LIHC patient survival was examined. By Kaplan‐Meier analysis, increased expression levels of mRNAsi were significantly associated with poor OS of LIHC patients (Figure 1B). Further, the clinical characteristics of mRNAsi in LIHC were analyzed. Patients with LIHC were divided into groups by age, sex, grade, and TNM stage, respectively (Figure 1C‐H); for which, the mRNAsi index was not associated with age (*P* = .787), gender (*P* = .121), or T (*P* = .053), N (*P* = .643), and M (*P* = .327) stage. However, there were significant differences in mRNAsi in different grades of LIHC (Figure 1E). Thus, mRNAsi expression was positively correlated with tumor grade in LIHC.

### 3.2 Identification of DEGs and screening of key genes related to mRNAsi

**FIGURE 2:**
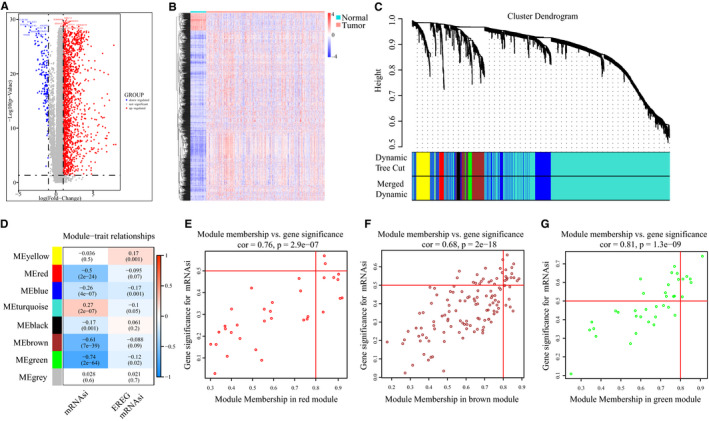
Screening of key genes related by mRNAsi. A, Volcano map of differentially expressed genes (DEGs); red represents upregulated genes, and blue indicates downregulated genes. B, Heatmap of DEGs. C, WGCNA analysis of DEGs. Branches with different colors correspond to eight different modules. D, Correlation analysis of the modules and clinical traits with mRNAsi or EREG‐mRNAsi. *P*‐values are shown. Scatter plot analysis of modules in the red (E), brown modules (F) and green (G)

To further explore the functional genes related to mRNAsi, the TCGA dataset was reanalyzed to filter and identify the DEGs between tumor and normal tissues (Figure 2A). In total, 1709 DEGs were identified, of which 1550 were upregulated, and 159 were downregulated (Figure 2B).

**FIGURE 3:**
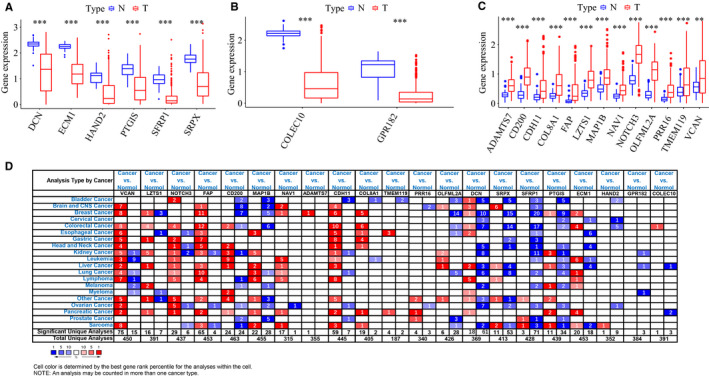
Expression of key genes related to mRNAsi. A, Comparison of gene expression levels in green (A), red (B), and brown (C) modules between normal and tumor samples. D, The mRNA expression patterns of 21 key genes in overall cancers in Oncomine database

To identify the biological key genes related to mRNAsi, we applied WGCNA to select the DEGs. For more precise analysis, outlier data were excluded, and the remaining 1709 DEGs were divided into modules by cluster analysis. A soft threshold (*β* = 3, scale‐free *R* ^2^ = .950) was used to guarantee a scale‐free network, which identified eight modules (Figure 2C). Next, whole gene expression levels of related modules were analyzed to find correlations between corresponding modules and mRNAsi in LIHC samples. The red, brown, and green modules exhibited negative correlations with mRNAsi, with correlations close to −0.5 (Figure 2D). Therefore, these three modules were chosen for further analyses, and 21 critical genes associated with mRNAsi were screened using the threshold cor. MM > 0.8 and cor. GS > 0.5. Among them, six genes (*DCN*, *ECM1*, *HAND2*, *PTGIS*, *SFRP1*, and *SRPX*) were in the green module, two genes (*COLEC10* and *GRP182*) in the red module, and 13 genes (*ADAMTS7*, *CD200*, *CDH11*, *COL8A1*, *FAP*, *LZTS1*, *MAP1B*, *NAV1*, *NOTCH3*, *OLFML2A*, *PRR16*, *TMEM119*, and *VCAN*) in the brown module, as shown in Figure 2E. We further mapped their expression tendency in normal and tumor tissues and found that the candidate genes in the red and green modules were downregulated in LIHC (Figure 3A,B). However, genes in the brown module were upregulated in LIHC (Figure 3C). In addition, we validated that genes in the brown module were also overexpressed in many other cancers, compared to normal samples, using the Oncomine dataset. Consistent with these findings, expression of genes in the red and green modules was greater in normal tissues in the Oncomine dataset (Figure 3D).

### 3.3 Co‐expression network of candidate genes

**FIGURE 4:**
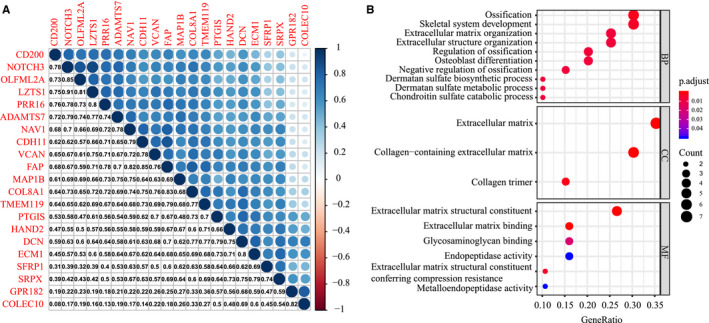
Co‐expression network of candidate genes. (A) Transcription‐level correlation analysis of 21 key genes among the three modules. (B) Bubble diagrams analysis of 21 key genes in LIHC

To explore relationships among these key genes, we performed co‐expression network analysis and found a strong correlation between candidate genes in their modules, with a correlation coefficient of at least 0.6. Among these modules, genes in the brown module showed no significant correlation with the red module, with the highest correlation coefficient of 0.36. However, there was a moderate correlation with genes in the green module, with a correlation coefficient range of 0.31‐0.77. In addition, the correlation coefficient between red and green modules was nearly 0.5 (Figure 4A). To further study the function of these candidate genes, Gene ontology (GO) term analyses were performed. Biological processes analysis showed enrichment of GO terms associated with ossification and dermatan sulfate. We also found in molecular functions analysis that extracellular matrix structural constituent, extracellular matrix binding, glycosaminoglycan binding, and endopeptidase activity were highly enriched in LIHC. Moreover, cellular component analysis showed that extracellular matrix, collagen‐containing extracellular matrix, and collagen trimmer were significantly regulated by mRNAsi factors in LIHC (Figure 4B).

### 3.4 Pathway and process enrichment analysis of key genes

**FIGURE 5:**
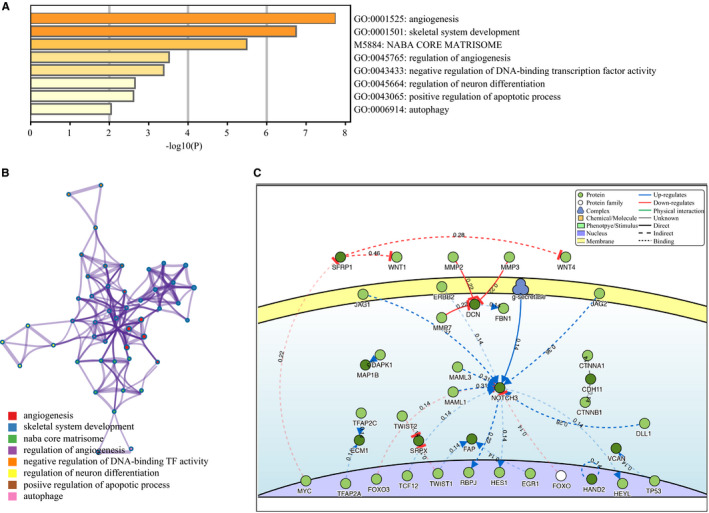
Functional analysis and causal relationship with proteins. A, KEGG functional analysis of 21 key genes in liver hepatocellular carcinoma (LIHC). B, Detailed net structure of key genes in LIHC (Metascape). C, Causal interaction analysis of 21 key genes in DisNor

Next, kyoto encyclopedia of genes and genomes (KEGG) pathway and process enrichment analysis were performed. The top 8 clusters with their representative enriched terms are shown in Figure 5A. Enriched terms across these candidate genes were identified for pathways involved in angiogenesis, negative regulation of DNA‐binding transcription factor activity, regulation of neuron differentiation, positive regulation of apoptotic process, and autophagy. Further, terms were selected with a similarity >0.3, and a network plot was constructed by Metascape (Figure 5B). As demonstrated in the KEGG pathway, angiogenesis and autophagic pathways were more enriched, suggesting that these pathways may be closely related to mRNAsi in LIHC.

### 3.5 Causal relationship with proteins

DisNor was used to reveal neighbors of these candidate genes by searching all interactions involving any of the seed entities and pruned nodes with degree‐one as the first neighbors’ data. Two proteins FAP and SRPX exhibited a close relationship with the TWIST protein. Furthermore, the level 3 data showed all signaling interactions without additional filtering, such as NOTCH3, TP53, FOXO2, FOXO3, TWIST, DCN, WNT1, WNT4, SFRP1, and MYC (Figure 5C). This indicated that the Wnt, Notch, and Hypoxia signaling pathways were closely associated with LIHC tumorigenesis.

## 4 DISCUSSION

Liver hepatocellular carcinoma is a comprehensive disease with high mortality and morbidity.^1^ It has been reported that CSCs play a key role in tumor development.^22^ Thus, therapeutic targeting of LIHC stem cells is of great importance. CSCs possess strong drug resistance and metastasis characteristics, and the regulatory mechanism of stem cell self‐renewal has not been fully elucidated. Therefore, targeted treatment of tumor stem cells may be a new strategy to combat tumor metastasis and recurrence. Here, mRNAsi scores were calculated as a factor involved in tumorigenesis, identifying critical genes related to CSC characteristics using WGCNA.

In the TCGA dataset of LIHC, the mRNAsi scores in tumor tissues were much higher than normal samples. In addition, the modified mRNAsi scores were closely related to the degree of differentiation of LIHC, such that lower differentiation status of the tumor corresponded to higher mRNAsi values. It is well known that undifferentiated malignant tumors can more easily induce malignant cancer cells in other tissues or organs, contribute to disease progression, and death.^23^ As such, CSCs are often resistant to chemotherapy and can escape the killing effect of drugs. Gain of stem/progenitor‐like cell features and loss of mature cell phenotypes may enhance tumor progression, which is consistent with the increased mRNAsi scores in LIHC. In this study, the mRNAsi index was assessed during progression after tumor development, demonstrating that higher mRNAsi scores were related to poorer OS in LIHC patients.

Weighted gene co‐expression network analysis was used to find co‐expression gene modules, explore the relationship between the gene network and phenotype, and identify key genes in the network. Among these eight modules, the red, brown, and green modules showed negative correlations with mRNAsi. Functional annotations of key genes associated with these significant modules showed enrichment in pathways involved in angiogenesis, differentiation, apoptotic process, and autophagy. Further, expression levels of these key genes showed obvious differences among the significant modules. In the red and green modules, these genes were significantly downregulated in LIHC tissues, while upregulated in the brown module. Functional analysis of genes in the red and green modules demonstrated participation in the negative regulation of ossification (GO:0030279) and apoptotic signaling pathway (GO:0097190), which might explain the over‐proliferative characteristics of tumor cells. Genes in the brown module were enriched in extracellular matrix organization (GO:00330198) and modulation of chemical synaptic transmission (GO:0050804), which might be closely related to metastasis and recurrence of LIHC.

In addition, we found that genes *ECM1*, *HAND2*, *PTGIS*, *SFRP1*, *SRPX*, *COLEC10*, and *GPR182* were significantly under‐expressed in LIHC in the Oncomine database, with the exception of *DCN*. Extracellular matrix protein 1 (ECM1) is expressed in the extracellular matrix of the liver, and knockout of ECM1 has been shown to lead to spontaneous and severe liver fibrosis in mice.^24^, ^25^ When the liver is in a pathological state, the function of hepatocytes is damaged, leading to decreased ECM1 protein production by hepatocytes, subsequently promoting the occurrence and development of liver fibrosis. *PTGIS*, member of the CYP450 family, is a prostacyclin synthase that can catalyze the conversion of PGH‐2 to PGI‐2. Expression of *PTGIS* has been shown to be decreased in the liver of mice with hepatic fibrosis compared to wild mice.^26^, ^27^ Further, expression of *SFRP1* (secreted frizzled‐related protein) is decreased and may play an important role in the development of hepatocellular carcinoma.^28^ Taken together, our findings indicate that the key genes in the red and green modules are closely associated with the development of LIHC.

Further, the Oncomine results demonstrated that the critical genes overexpressed in LIHC were also highly expressed in a variety of other cancers, including breast cancer, colorectal cancer, kidney cancer, lung cancer, leukemia, and lymphoma, and could be potential therapeutic targets for LIHC. Specifically, 9 of 13 genes (*VCAN*, *LZTS1*, *NOTCH3*, *FAP*, *CD200*, *MAP1B*, *NAV1*, *CDH11*, and *OLFML2A*) were identified in at least 1% of the gene bank for LIHC. *VCAN* is related to the maintenance and differentiation of stem cells. It is involved in the pluripotency regulatory pathway as well as the Wnt and Notch signaling pathways. Further, *VCAN* mutation may lead to phenotypic differentiation of hepatocellular carcinoma and cholangiocarcinoma in mixed cancer.^29^ *NOTCH3* is a highly conserved member of the *NOTCH* family. Recent studies have shown that the *NOTCH3* signaling pathway is closely related to the occurrence and development of liver cancer.^30^, ^31^ Further, FAP and MMP‐1 have been shown to be positively correlated with the degree of liver fibrosis in mice. Multivariate regression analysis showed that FAP‐1 expression, vascular invasion, tumor size, and TNM stage were independent risk factors for prognosis of LIHC patients.^32^ *OLFML2A* is highly expressed in LIHC, and knockout of *OLFML2A* has been shown to inhibit growth and proliferation and promote apoptosis of LIHC cells.^33^

## 5 CONCLUSIONS

In conclusion, we found a novel biomarker mRNAsi to predict the prognosis of LIHC. In addition, 21 critical genes were found that played important roles in LIHC stem cell maintenance using WGCNA analysis. Functional analysis of these key genes showed enrichment in the pluripotency regulatory pathway as well as Wnt and Notch signaling pathways. Further, the apoptotic and autophagy signaling pathways were inhibited in LIHC. Therefore, these genes could be potential therapeutic targets for stemness inhibition of LIHC. Further biological and basic studies are needed to validate our findings.

## CONFLICT OF INTEREST

The authors declare no conflict of interest.

## AUTHOR CONTRIBUTIONS

YD and LC designed the concept and experiments; KB, SH, LS, and WW performed the experiments; KB, SH, SL, QZ, and LL collected the data and did the analysis. KB and SH prepared the manuscript draft. YD and LC revised the manuscript. All the authors approved the final proof.

## Supporting information

Figure S1

## Data Availability

The datasets used and/or analyzed during the current study are available from the corresponding author on reasonable request.
