# Supplementary figures and images for: Identification of cancer stem cell characteristics in liver hepatocellular carcinoma by WGCNA analysis of transcriptome stemness index
Source: Cancer Med. 2020 Apr 20;9(12):4290–8. doi: 10.1002/cam4.3047 (PMC7300398; doi:10.1002/cam4.3047)

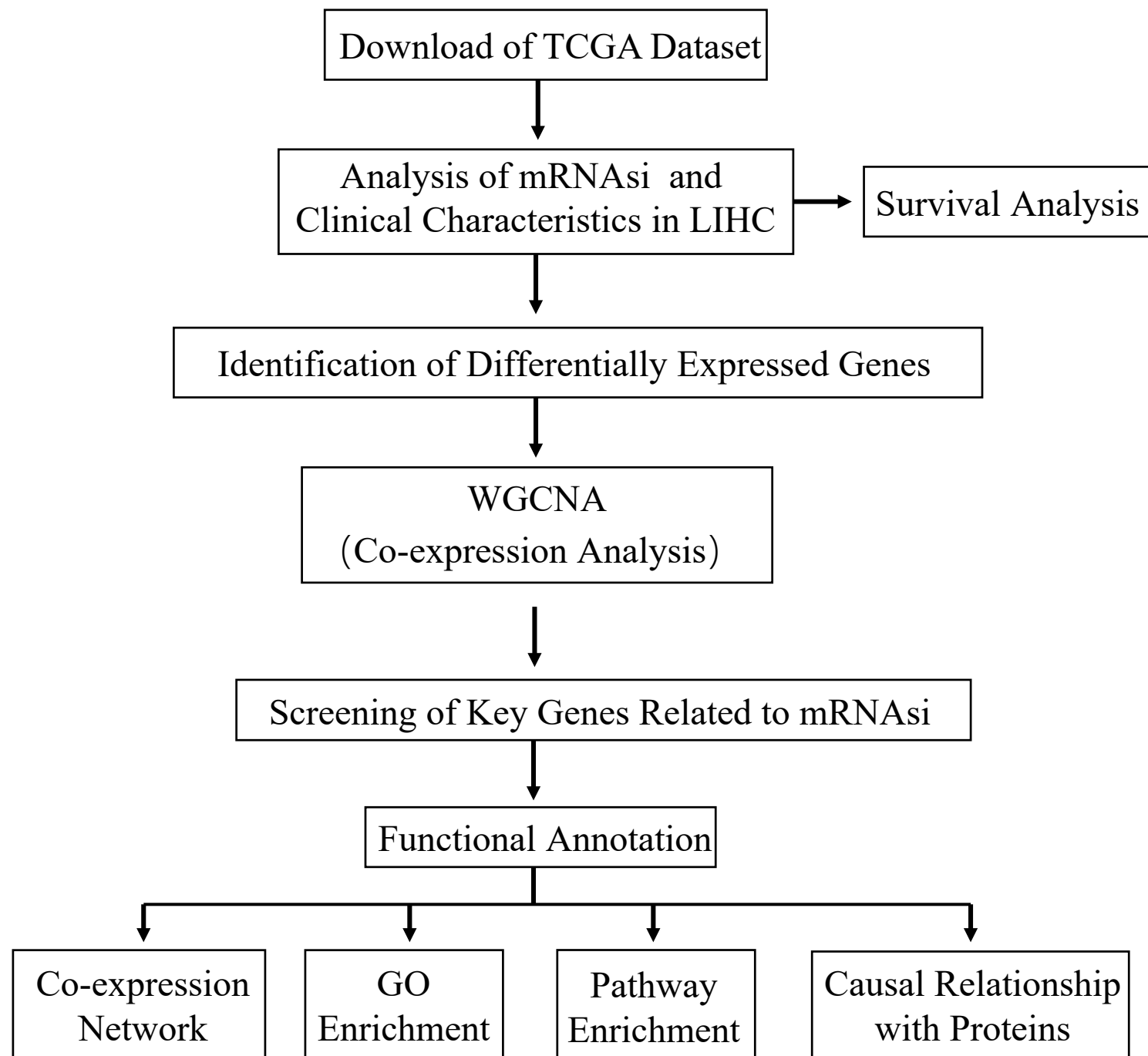

Supplement: Supplementary file 1 — Figure S1 [file CAM4-9-4290-s001.pdf]
